# The dose-response association between LEAP 1000 and birthweight – no clear mechanisms: a structural equation modeling approach

**DOI:** 10.1186/s12884-023-05707-1

**Published:** 2023-05-19

**Authors:** Sarah Quinones, Shao Lin, Lili Tian, Pauline Mendola, Jacob Novignon, Clement Adamba, Tia Palermo

**Affiliations:** 1 Department of Epidemiology and Environmental Health, School of Public Health and Health Professions, University at Buffalo, State University of New York, Buffalo, NY 14214 USA; 2 Department of Environmental Health Sciences, One University Place, 212D University at Albany, State University of New York, Rensselaer, NY 12144 USA; 3 Department of Epidemiology and Biostatistics, One University Place, 212D University at Albany, State University of New York, Rensselaer, NY 12144 USA; 4 Department of Biostatistics, State University of New York, 717 Kimball Tower University at Buffalo, Buffalo, NY 14214 USA; 5 Department of Economics, Kwame Nkrumah University of Science and Technology, Kumasi, Ghana; 6 Institute of Statistical, Social and Economic Research, University of Ghana-Legon, P.O. Box LG 74, Legon-Accra, Ghana

**Keywords:** LEAP 1000, Birthweight, Ghana, Empowerment, Antenatal care, Food insecurity, SEM, Cash transfers

## Abstract

**Background:**

Birthweight is an important indicator of maternal and fetal health globally. The multifactorial origins of birthweight suggest holistic programs that target biological and social risk factors have great potential to improve birthweight. In this study, we examine the dose-response association of exposure to an unconditional cash transfer program before delivery with birthweight and explore the potential mediators of the association.

**Methods:**

Data for this study come from the Livelihood Empowerment Against Poverty (LEAP) 1000 impact evaluation conducted between 2015 and 2017 among a panel sample of 2,331 pregnant and lactating women living in rural households of Northern Ghana. The LEAP 1000 program provided bi-monthly cash transfers and premium fee waivers to enroll in the National Health Insurance Scheme (NHIS). We used adjusted and unadjusted linear and logistic regression models to estimate the associations of months of LEAP 1000 exposure before delivery with birthweight and low birthweight, respectively. We used covariate-adjusted structural equation models (SEM) to examine mediation of the LEAP 1000 dose-response association with birthweight by household food insecurity and maternal-level (agency, NHIS enrollment, and antenatal care) factors.

**Results:**

Our study included a sample of 1,439 infants with complete information on birthweight and date of birth. Nine percent of infants (N = 129) were exposed to LEAP 1000 before delivery. A 1-month increase in exposure to LEAP 1000 before delivery was associated with a 9-gram increase in birthweight and 7% reduced odds of low birthweight, on average, in adjusted models. We found no mediation effect by household food insecurity, NHIS enrollment, women’s agency, or antenatal care visits.

**Conclusions:**

LEAP 1000 cash transfer exposure before delivery was positively associated with birthweight, though we did not find any mediation by household- or maternal-level factors. The results of our mediation analyses may serve to inform program operations and improve targeting and programming to optimize health and well-being among this population.

**Trial Registration:**

The evaluation is registered in the International Initiative for Impact Evaluation’s (3ie) Registry for International Development Impact Evaluations (RIDIESTUDY- ID-55942496d53af) and in the Pan African Clinical Trial Registry (PACTR202110669615387).

**Supplementary Information:**

The online version contains supplementary material available at 10.1186/s12884-023-05707-1.

## Background

Infant birthweight is a critical metric of maternal and fetal health and a key predictor of child and adult health outcomes globally. Low birthweight (LBW; < 2,500 g) infants have increased risk of morbidity, mortality, malnutrition, and chronic disease throughout the life course compared to healthy weight infants [1–4]. Mothers born LBW are more likely to give birth to a LBW infant, suggesting intergenerational persistence of impaired fetal health and development [5]. Despite global reductions of LBW, prevalence remains high in African countries with 14% prevalence in sub-Saharan Africa in 2015 [6]. Further, average birthweight has trended downward in Africa in the 21st century [7]. Taken together, these trends suggest a need for interventions to improve birthweight outcomes in this region.

LBW is a multifactorial birth outcome which arises from preterm birth (PTB; delivery before 37 completed weeks of gestation), intrauterine growth restriction (IUGR; infant growth did not reach full biological potential), or a combination of the two. Prevention of LBW relies on comprehensive interventions that target risks to the health of the mother and the developing fetus [8, 9]. The multifactorial origins of LBW risk present many opportunities for intervention and risk reduction. Comprehensive interventions that target the myriad risk factors of reduced birthweight and increased LBW may serve as cost-effective approaches to improved health, though evidence on such interventions is lacking. Most birthweight interventions have focused primarily on nutrition during pregnancy [9]. However, there are several other predictors of birthweight, many of which are poverty-related, worth targeting for LBW risk reduction in low-resource populations of Africa.

Social protection, defined as “the set of policies and programs aimed at preventing or protecting all people against poverty, vulnerability and social exclusion throughout their lifecycle, with a particular emphasis towards vulnerable groups,” [10] is a potential cost-effective intervention for LBW risk reduction. Specifically, cash transfers (CTs), whereby recipients receive scheduled and predictable amounts of cash based on poverty or other criteria, have been associated with reduced LBW risk in various contexts [11]. However, there is a dearth of evidence on the (1) dose-response associations between CT program participation and birthweight and (2) mediators by which CTs, specifically unconditional CTs (UCTs), improve birthweight. UCTs require no actions on the part of the recipients to be eligible for payments. In contrast, the literature includes studies that evaluate conditional CTs (CCTs) [11], which require that beneficiaries adhere to certain behaviors, such as maternal and child healthcare visits or school enrollment and attendance, to receive payments. A recently published study was the first to identify positive impacts of a UCT on birthweight and LBW in Africa [12].

This study seeks to contribute to this evidence base by examining (1) the association between months of exposure to a UCT program before delivery and birthweight and (2) the pathways through which these associations materialize. We hypothesize that a UCT program coupled with health insurance enrollment targeted to pregnant women will increase birthweight and decrease LBW risk among infants through the pathways of household food security, antenatal care (ANC), women’s agency, and health insurance [13–15].

## Materials and methods

### Livelihood empowerment against poverty (LEAP) 1000 program

In 2008, the Ministry of Gender, Children and Social Protection (MoGCSP; Government of Ghana) implemented LEAP, its flagship national social protection program. The purpose of LEAP was to reduce poverty in the short-term and improve human capital development in the long-term [16]. To achieve these objectives, LEAP provided cash payments to households living in extreme poverty with a household member from a vulnerable demographic group (i.e., orphan or vulnerable child, elderly person, or a person with a severe disability). Then, in 2011, the National Health Insurance Authority (NHIA) and the MoGCSP collaborated to enroll LEAP beneficiaries into the National Health Insurance Scheme (NHIS) under the NHIA ‘indigent’ exemption, which waives NHIS enrolment and other fees, including card processing, premiums, and renewals. As of 2017, LEAP reached more than 200,000 households in Ghana, and as of 2022, it now reaches 550,000 households nationally.

In 2015, a pilot program within LEAP – LEAP 1000 - expanded program eligibility to pregnant and lactating women living in extremely impoverished, rural households in districts of Northern and Upper East Ghana. The objective of the LEAP 1000 pilot program was to reduce malnutrition and stunting. To achieve this objective, LEAP 1000 aimed to target children in the first 1,000 days of their lives (i.e., from conception to age 2 years). Using a multi-stage targeting approach, communities in 10 districts of Northern and Upper East Ghana were identified using district-level poverty rankings and then households in the poorest communities (with priority given to those not already covered by LEAP) were selected based on proxy means test (PMT) scores that served as measures of household poverty status. PMTs were administered to households containing women of reproductive age (15–49 years) who were eligible if (1) they were pregnant or (2) they had a child 12 months of age or younger and could present health facility documentation to confirm their status.

The effectiveness of the LEAP 1000 pilot program was tested in an impact evaluation, led collaboratively by the UNICEF Office of Research – Innocenti, the University of North Carolina at Chapel Hill, the Institute for Social, Statistical, and Economic Research (ISSER), and the Navrongo Health Research Centre. The impact evaluation was conducted between 2015 and 2017 in 5 of the 10 initial districts where LEAP 1000 was piloted (Bongo, East Mamprusi, Garu-Tempane, Karaga, and Yendi). Power calculations run for the original impact evaluation found that program impacts on the primary outcomes of interest (stunting, wasting, and underweight) could be observed with a sample size of 2,500 households (1,250 comparison and 1,250 treatment). However, these power calculations were not conducted with secondary outcomes, such as LBW, in mind. The impact evaluation sample selection was inspired by a Regression Discontinuity Design (RDD) identification strategy that leveraged a PMT score threshold to select a census of 1,250 comparison households just above the threshold and 1,250 treatment households just below the threshold for interviews to maximize comparability between groups. At baseline, 2,497 eligible households (1,235 comparison and 1,262 treatment) were included. By endline, 6% of baseline households were lost to follow-up, leading to panel sample of 2,331 households (1,146 comparison and 1,185 treatment) used for the impact evaluation and which serves as the sample for this secondary data analysis.

### Data collection

Household questionnaires were administered to household heads and/or LEAP 1000 eligible women (one per household) by trained enumerators at baseline (July – September 2015) and endline (June to August 2017). Topics covered by the household questionnaire included housing conditions and WASH, food security, time use and employment, productive livelihoods, non-farm enterprises, reproductive health, and household consumption. Topics covered by the LEAP 1000 beneficiary questionnaire included birth history, contraception and fertility preferences, women’s agency, stress and preferences, nutrition and feeding knowledge, and intimate partner violence. Lastly, LEAP 1000 beneficiaries were asked about their children in the questionnaires using the following topics: maternal and newborn health, child health, immunizations, child nutrition and feeding, birth registration and child development, and anthropometry.

### Measures

Our dependent variables included infant birthweight (measured in kilograms; from maternal recall and records on health cards) and LBW (birthweight < 2.5 kg). The independent variable was months of LEAP 1000 treatment received before infant delivery, which was calculated based on the difference in months between infant birth date and LEAP 1000 implementation (September 2015). All comparison infants and treatment infants born before program implementation were classified as having zero months of exposure before delivery.

**Fig. 1:**
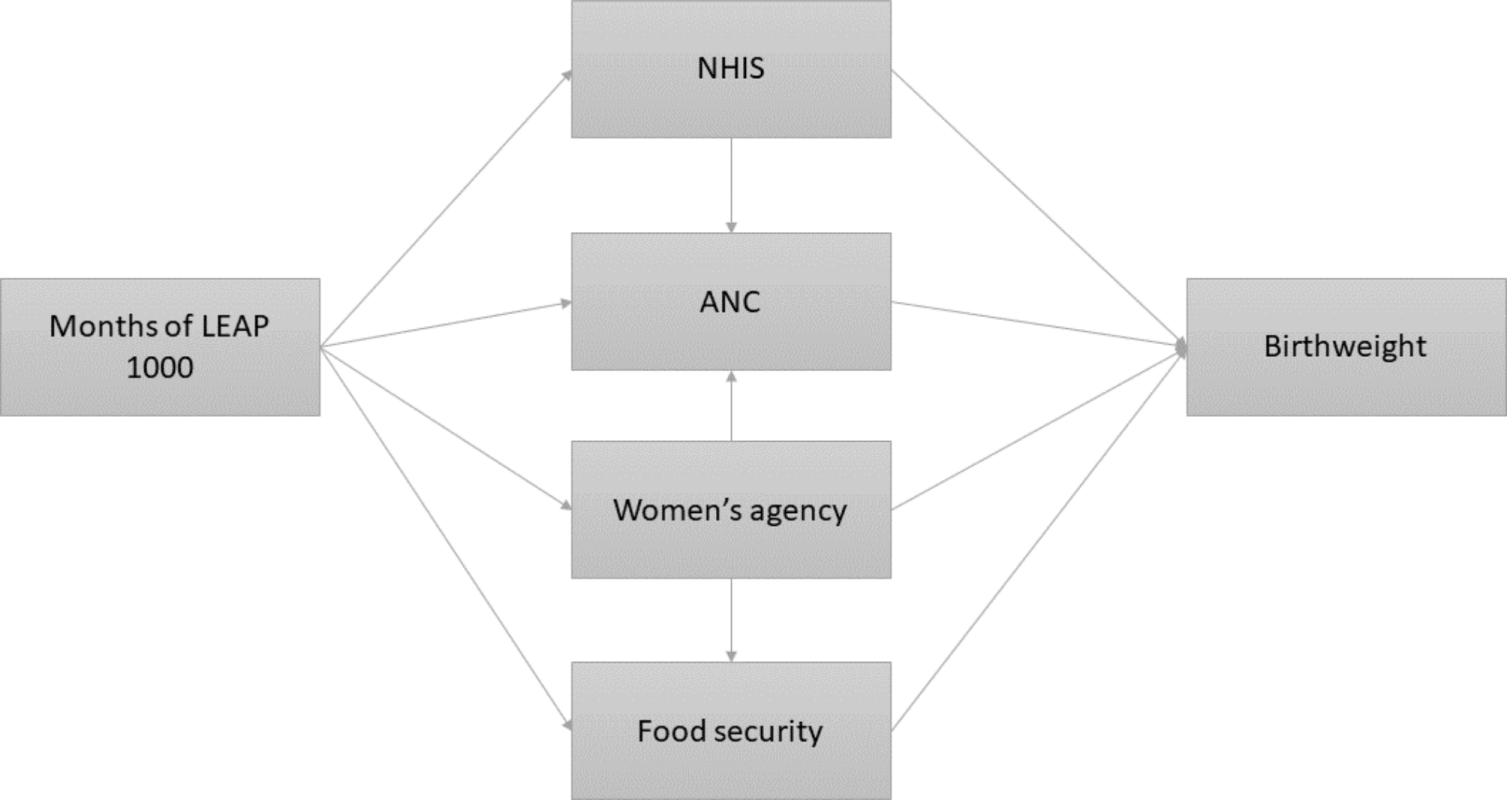
Hypothesized pathways between months of LEAP 1000 exposure before infant delivery and birthweight

Potential mediators evaluated in this study are shown in Fig. 1. These mediators were selected based on the LEAP 1000 conceptual framework (Supplementary Fig. 1) and the results of the LEAP 1000 impact evaluation showing positive impacts on these variables [13]. Potential mediators included current NHIS enrollment (a current and valid NHIS card observed by the enumerator), self-reported ANC visits with a skilled provider, number of ANC visits during pregnancy, and number of meals reported per day. Household food insecurity score was calculated based on the sums of the following indicators: (1) the household head reported worrying that their household didn’t have enough food more than once in the past 4 weeks (0: Never; 2: Rarely; 3: Sometimes; 4: Often) and (2) the household reported that a household member went an entire day and night without food more than once in the past 4 weeks (0–4). Additionally, women’s agency was included as a potential mediator, informed by the literature that suggests CTs improve agency and that agency is a salient predictor of maternal and child health outcomes [17–21]. The definition of women’s agency was based on the sum of the following indicators [22]: In the past 12 months, how often did you feel that (a) Your life is determined by your own actions; (b) You have the power to make important decisions that change the course of your own life; (c) You have the power to make important decisions that change the wellbeing of your children; (d) You have the power to make important decisions that change the wellbeing of your household; (e) You are capable of protecting your own interests within your household; and (f) You are capable of protecting your own interests outside of your household. Reponses were on a scale of 1 (never) to 5 (very often/always). Prior to summing these indicators for a total women’s agency score, each indicator was dichotomized as classified as 1 if at least sometimes and classified as 0 otherwise for a total score range of 0–6.

### Statistical analysis

Bivariate analyses were conducted to test any differences in household-, maternal-, and infant-level characteristics between infants who did not receive any LEAP 1000 treatment before delivery (either comparison infants or treatment infants born before LEAP 1000 implementation) and those who received 1 or more months of LEAP 1000 treatment before delivery. We present bivariate analyses using logistic regression for dichotomous outcomes and linear regression for continuous outcomes, adjusted for PMT score. To test the dose-response relationship between the number of months of LEAP 1000 exposure before delivery and birthweight, we estimated coefficients and confidence intervals (CI) using crude and adjusted linear regression models. Crude and adjusted logistic regression models were used to estimate the associations between months of LEAP 1000 and LBW with odds ratios (OR) and 95% CI.

Model covariates were selected if differences were statistically significant across categories of months of LEAP 1000 exposure during pregnancy (p < 0.1). Covariates included total number of children under age 5 in the household, parity, household has an improved lighting source, and district of residence. Given that sample selection was informed by the threshold based on PMT score distributions, we also adjusted for PMT score. Additionally, to adjust for potential time trends, month and year of birth were included as covariates in final models.

Mediation analysis was conducted using an adjusted generalized Structural Equation Model (SEM). SEM presents the direct and indirect of months of LEAP 1000 on birthweight through ANC, NHIS, women’s agency, and household food insecurity measured at endline to ensure temporality in these associations, while adjusting for all covariates outlined above. SE were clustered at the household-level in the SEM and regression models. All analyses were conducted using Stata version 16 (g*sem* command for mediation analysis [*nlcom* command for individual and total effects]) [23]. We describe results as statistically significant at an alpha less than 5%, though in mediation analyses we highlight results at a p-value of 10% using boldface.

### Sensitivity analyses

We ran generalized SEM with LBW as a dichotomous dependent variable as a test of our main results for continuous birthweight. These models were adjusted for the same covariates as in the main analysis, SEs were clustered at the household level, and the generalized model was specified with a ‘binomial’ family and a ‘logit’ link function. These models estimate the change in log odds of LBW with 95% CI in response to changes in the independent variable (months of LEAP 1000) and the mediators in the model.

## Results

**Fig. 2:**
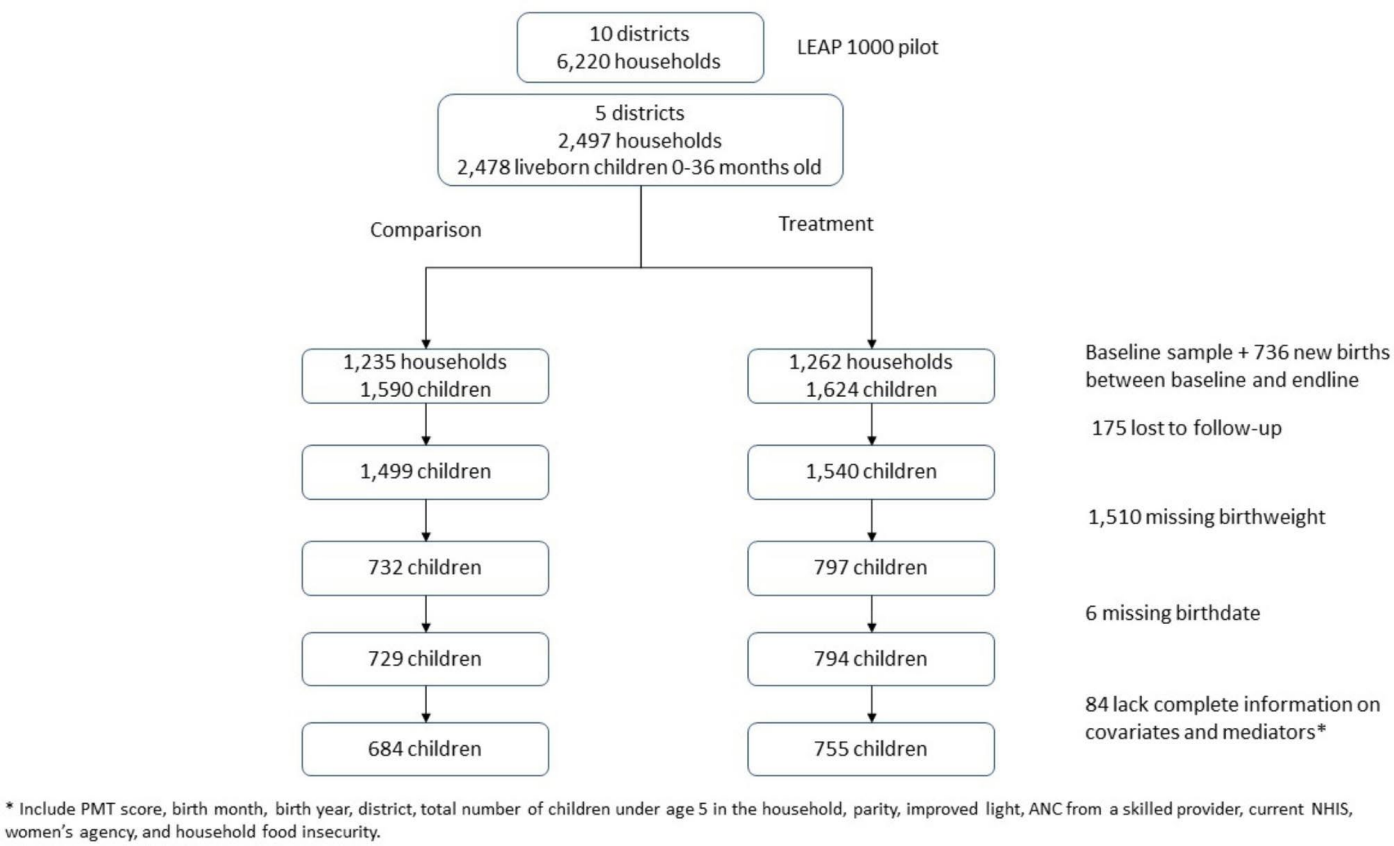
CONSORT diagram of LEAP 1000 impact evaluation and study sample selection

The final analytic sample for this study included 1,439 infants born to women who were part of the LEAP 1000 impact evaluation (treatment and comparison groups) from 2015 to 2017 with complete information on birthweight (~ 50% of the full sample of infants), birth date, and other model mediators and covariates (Fig. 2) [13].

Among the 1,439 infants in the analytic sample, 129 (9%) were exposed to at least one month of LEAP 1000 before delivery. Comparisons of household-, maternal, and infant-level characteristics by number of months of treatment (adjusted for PMT score) are shown in Table 1. Comparison and treatment infants born before program implementation were generally comparable to infants who received one or more months of LEAP 1000. Infants who received one or more months of LEAP 1000 were less likely to be born in the rainy season (57%) than those who did not receive LEAP 1000 before delivery (66%; p = 0.009). A lower proportion of infants treated before delivery resided in Bongo (19%) than those who did not receive LEAP 1000 before delivery (26%; p = 0.028), though a higher proportion resided in East Mamprusi (47 vs. 41%, respectively; p = 0.054). Higher maternal parity, number of children under the age of 5 years in the household, and improved lighting sources were observed among the infants treated by LEAP 1000 before delivery compared to those not treated. Current NHIS enrollment was observed to be lower among women who received at least 1 months of LEAP 1000 treatment (66%) than those who did not (72%; p = 0.008). Women’s agency was significantly lower among those not exposed to LEAP 1000 before delivery (3.02 ± 1.95) compared to those with at least 1 month of LEAP 1000 exposure before delivery (4.11 ± 1.79; p < 0.001). Household food insecurity score was significantly lower among those exposed before delivery (0.60 ± 0.52) compared to those who were not (3.79 ± 1.99; p < 0.001). We observed no significant differences by category of LEAP 1000 exposure before delivery for any measure of ANC.

**Table 1:** Descriptive statistics of sample characteristics by number of months of LEAP 1000 before delivery among a sample of infants with complete birthweight born to women receiving the LEAP 1000 cash transfer (N = 1,439)

	Number of months of LEAP 1000 Mean ± SD or N (%)		
	0	1+	p-value^#^
**Household-level**			
Number of children under 5 in household	1.76 ± 0.71	2.09 ± 0.62	**< 0.001**
Household head married	1,245 (95)	125 (97)	0.275
Female household head	133 (10)	8 (6)	0.132
Age of household head	39.3 ± 13	41.4 ± 12.2	0.457
Household has improved lighting source	406 (31)	52 (40)	**0.004**
Household food insecurity score (0–8)	3.79 ± 1.99	0.60 ± 0.52	**< 0.001**
**Maternal-level**			
Number of meals consumed per day	2.6 ± 0.6	2.6 ± 0.58	0.851
Current NHIS enrollment	948 (72)	85 (66)	**0.008**
Mother’s age	29.2 ± 6.52	30.8 ± 6.21	0.117
Women’s agency (0–6)	3.02 ± 1.95	4.11 ± 1.79	**< 0.001**
Parity (Total number of live births)	3.67 ± 1.96	4.78 ± 2.17	**< 0.001**
Sought ANC from skilled provider	1,218 (93)	120 (93)	0.291
Number of ANC visits	5.89 ± 1.73	5.92 ± 1.66	0.897
Infant-level			
Singleton	1,263 (96)	127 (98)	0.164
Delivered in a health facility	1,220 (93)	117 (91)	0.325
Infant birthweight (kg)	3.01 ± 0.46	3.07 ± 0.46	0.195
Infant low birth weight (< 2.5 kg)	95 (7)	8 (8)	0.501
Infant female	655 (50)	56 (43)	0.177
Infant born during the rainy season (March - Sept)	868 (66)	73 (57)	**0.009**
**District**			
East Mamprusi	537 (41)	61 (47)	**0.054**
Karaga	60 (5)	6 (5)	0.810
Yendi	104 (8)	10 (8)	0.973
Bongo	346 (26)	24 (19)	**0.028**
Garu-Tempane	263 (20)	28 (22)	0.959
*N*	1,310	129	

ANC: Antenatal care; NHIS: National Health Insurance Scheme; PMT: Proxy means test; SD: Standard deviation

^#^ Tests of significance conducted using logistic and linear regression models adjusted for PMT score for dichotomous and continuous variables, respectively

The unadjusted and adjusted associations between months of LEAP 1000 treatment before delivery and birthweight are presented in Table 2. In this sample, birthweight was normally distributed with normally distributed errors when regressed with months of LEAP 1000 exposure before delivery. On average, a 1-month increase in LEAP 1000 exposure before delivery was associated with a 9-gram increase in infant birthweight in the adjusted model (p = 0.015). Also, increased parity was marginally associated with increased birthweight (p = 0.054), while later year of birth was associated with decreased birthweight (p = 0.027), suggesting a negative trend in birthweight over time in this sample.

**Table 2:** Unadjusted and adjusted associations between number of months of LEAP 1000 treatment before delivery and birthweight among a sample of 1,439 Ghanaian infants; 2015–2017

	Infant birthweight (kg) β (95% CI) p-value	
	Unadjusted^a^	Adjusted^b^
Months of LEAP 1000 Treatment	0.005 (-0.001–0.011)	0.009 (0.002–0.017)
	0.095	**0.015**
Household-level		
Food insecurity score (0–8)		-0.002 (-0.018–0.014)
		0.763
Improved lighting source		0.031 (-0.028–0.089)
		0.302
Number of children under 5 in household		-0.007 (-0.046–0.032)
		0.729
PMT score	0.006 (-0.305–0.316)	0.111 (-0.209–0.431)
	0.971	0.495
Maternal-level		
Parity		0.014 (-0.000–0.027)
		0.054
Current NHIS enrollment		0.037 (-0.020–0.094)
		0.199
Infant-level		
Month of birth		-0.004 (-0.013–0.005)
		0.383
Year of birth		-0.041 (-0.077 - -0.005)
		0.027
Infant born during the rainy season (March - Sept)		0.005 (-0.050–0.060)
		0.858
District [ref: East Mamprusi]		
Karaga		-0.029 (-0.172–0.113)
		0.687
Yendi		0.036 (-0.057–0.130)
		0.447
Bongo		-0.018 (-0.082–0.047)
		0.597
Garu-Tempane		-0.003 (-0.072–0.067)
		0.940
*N*	1,439	

PMT: proxy means test; NHIS: National Health Insurance Scheme. ^a^Model adjusted for PMT score; ^b^Model adjusted for PMT score, parity, improved lighting source in household, number of children under 5 years old in the household, district of residence, year of birth, infant born in the rainy season, current NHIS enrollment, household food insecurity score, and month of birth. Standard errors clustered at household level

In Table 3, we present the logistic regression estimates of the unadjusted and adjusted ORs and 95% CI for the association between months of LEAP 1000 before delivery and LBW. A 1-month increase in LEAP 1000 exposure before delivery was associated with 7% reduced odds of LBW in the adjusted model (p = 0.024). Increasing PMT score was associated with large reductions in odds of LBW (OR = 0.07; 95% CI: (0.005–0.910); p = 0.042). Current enrollment in the NHIS was marginally associated with reduced odds of LBW (p = 0.068) and living in Bongo versus East Mamprusi was associated with 50% reduced odds of LBW (p = 0.015).

**Table 3:** Unadjusted and adjusted associations between number of months of LEAP 1000 treatment before delivery and LBW among a sample of 1,439 Ghanaian infants; 2015–2017. PMT: proxy means test; LBW: Low birthweight; NHIS: National Health Insurance Scheme ^a^Model adjusted for PMT score; ^b^Model adjusted for PMT score, parity, improved lighting source in household, number of children under 5 years old in the household, district of residence, year of birth, infant born in the rainy season, current NHIS enrollment, household food insecurity score, and month of birth. Standard errors clustered at household level

	Low birthweight OR (95% CI) p-value	
	Unadjusted^a^	Adjusted^b^
Months of LEAP 1000 Treatment	0.960 (0.907–1.017)	0.928 (0.869–0.990)
	0.164	**0.024**
Household-level		
Food insecurity score (0–8)		0.899 (0.783–1.032)
		0.130
Improved lighting source		0.842 (0.536–1.323)
		0.456
Number of children under 5 in household		1.023 (0.773–1.352)
		0.875
PMT score	0.220 (0.016–2.992)	0.068 (0.005–0.910)
	0.256	**0.042**
Maternal-level		
Parity		0.911 (0.801–1.037)
		0.157
Current NHIS enrollment		0.657 (0.419–1.032)
		0.068
Infant-level		
Month of birth		0.979 (0.906–1.058)
		0.593
Year of birth		1.068 (0.817–1.395)
		0.632
Infant born during the rainy season (March - Sept)		1.204 (0.752–1.928)
		0.439
District [ref: East Mamprusi]		
Karaga		1.180 (0.425–3.275)
		0.750
Yendi		0.679 (0.291–1.581)
		0.369
Bongo		0.495 (0.281–0.873)
		**0.015**
Garu-Tempane		0.541 (0.279–1.048)
		0.069
*N*	1,439	

Table 4 presents the independent variable-mediator and mediator-dependent variable associations estimated using adjusted linear regression models for months of LEAP 1000 exposure before delivery, potential household- and maternal-level mediators, and birthweight, respectively. Increasing months of LEAP 1000 exposure before delivery was associated with a significant reduction in household food insecurity score (β=-0.068; 95% CI: [-0.085, -0.05]; p < 0.001). We also observed a marginal improvement in women’s agency in response to increased number of months of LEAP 1000 before delivery (β = 0.023; 95% CI: [-0.004, 0.05]; p = 0.1). No other associations were statistically significant.

**Table 4:** Adjusted associations between mediators, months of LEAP 1000 before delivery, and birthweight; N = 1,439

	Independent variable - mediator Months of LEAP 1000 before delivery		Mediator – dependent variable Birthweight (kg)	
	**β coefficient (95% CI)**	**p-value**	**β coefficient (95% CI)**	**p-value**
Current NHIS enrollment	0.0002 (-0.006, 0.007)	0.961	0.033 (-0.023, 0.089)	0.253
Number of ANC visits	-0.007 (-0.032, 0.017)	0.548	-0.006 (-0.02, 0.008)	0.42
ANC from a skilled provider	-0.001 (-0.005, 0.003)	0.521	0.009 (-0.093, 0.112)	0.860
Number of meals consumed per day	-0.003 (-0.011, 0.006)	0.572	0.013 (-0.033, 0.059)	0.590
Food insecurity score (0–8)	-0.068 (-0.085, -0.05)	**< 0.001**	-0.001 (-0.016, 0.015)	0.948
Women’s agency score (0–6)	0.023 (-0.004, 0.05)	0.1	0.009 (-0.003, 0.022)	0.154

ANC: Antenatal care; PMT: Proxy means test. All regressions are adjusted for PMT score, month and year of birth, district of residence, total number of children under the age of 5 in the household, parity, improved source of lighting in the household, and season of birth (rainy v. dry). Standard errors were clustered at the household level

Table 5 is organized to show the independent and mediator variables estimated in the SEM in column (1), the direct effects of each variable on birthweight in column (2), the indirect effects of months of LEAP 1000 through all mediators combined and each mediator individually in column (3), and the percent due to mediation (indirect/total effect) in column (4). In the SEM, months of LEAP 1000 exposure before delivery had significant direct effects on birthweight in the adjusted models (β = 0.01; p = 0.006). Mediation through all mediating variables accounted for 1% of the effect of months of LEAP 1000 on birthweight. Women’s agency did not have any significant direct or indirect effects on birthweight, though mediation accounted for 2% of the adjusted LEAP 1000-birthweight association. Similarly, no statistically significant direct or indirect effects were observed for household food insecurity, NHIS enrollment, or ANC, though household food insecurity accounted for 8% of the LEAP 1000-birthweight association.

**Table 5:** Adjusted mediation effect of maternal and household-level characteristics on the association between months of LEAP 1000 treatment before delivery and birthweight among the sample of 1,439 infants

Variable (1)	Direct effect (2)	Indirect effect (3)	Percent due to mediation (4)	
Months of LEAP 1000	**0.01 (0.003, 0.018)** **0.006**	0.0001 (-0.001, 0.001) 0.808	0.0001/0.01 = 1%	
Women’s agency	0.009 (-0.004, 0.021) 0.188	0.0002 (-0.0002, 0.001) 0.298	0.0002/0.009 = 2.2%	
Household food insecurity score (0–8)	0.001 (-0.015, 0.017) 0.946	-0.00004 (-0.001, 0.001) 0.946	0.00004/0.0005 = 8%	
Current NHIS enrollment	0.032 (-0.024, 0.088) 0.263	5.15e-06 (-0.0002, 0.0002) 0.961	0.000005/0.03 = < 1%	
ANC from a skilled provider	0.012 (-0.088, 0.113) 0.811	-0.00002 (-0.0001, 0.0001) 0.807	0.00002/0.01 = < 1%	

ANC: Antenatal care; LEAP 1000: Livelihood Empowerment Against Poverty 1000 program; NHIS: National Health Insurance Scheme; PMT: Proxy means test. Models adjusted for PMT score, parity, improved source of lighting in the household, number of children under 5 years old in the household, district of residence, year of birth, infant born in the rainy season, and month of birth. Standard errors clustered at household level. **Boldface results are those with a p-value less than 10%**

Mediated effects of LEAP 1000 of LBW by the same set of mediators are presented in Supplementary Table 1. The overall direct effect of each month of LEAP 1000 before delivery on LBW was statistically significant (-0.084; 95% CI: [-0.151, -0.018]; p = 0.013) and mediation by these factors accounted for 7.7% of the total effect. Current NHIS enrollment had a marginally significantly negative association with LBW (-0.407; 95% CI: [-0.849, 0.036]; p = 0.072) but did not mediate the association between months of LEAP 1000 and birthweight. Similar to the findings in Table 5, no mediating effects were observed for current NHIS, ANC, women’s agency or household food insecurity, though the latter accounted for 7.6% of the association between months of LEAP 1000 and LBW.

## Discussion

We found a 9-gram increase in average birthweight and 7% reduced odds of LBW for each additional month of LEAP 1000 exposure before delivery in adjusted linear and logistic regression models, respectively. These findings were confirmed in the SEM models with a 10-gram increase in birthweight and log odds of LBW reduced by 8.4 in response to a 1-month increase in exposure to LEAP 1000 before delivery. We observed no mediating effect of ANC, current NHIS enrollment, women’s agency, or household food insecurity on these associations.

The evidence that CTs improve birthweight and reduce LBW risk is limited in general, and virtually nonexistent in Africa [11]. A previous study by our team was the first to examine whether a UCT in Africa impacted birthweight and LBW [12]. Saville and colleagues found that a Participatory Learning and Action women’s group with food transfers increased average birthweight by 78 g compared to a control group in Nepal [24]. Barber and Gertler found that Mexico’s *Oportunidades* CT program increased average birthweight by 102–127 g and decreased LBW by 4.4–4.6% points [25, 26]. In Colombia, Attanasio and colleagues found a 578-gram increase in the birthweight of urban infants born to women who participated in *Familias en Accion* CT program. And, Amarante and colleagues found the *PANES* CT program in Uruguay to increase average birthweight by 31 g and decrease LBW by 1.9–2.5% points [27]. A review by Glassman and colleagues included myriad studies from 8 countries that examined CT impacts on maternal and neonatal health and found improved prenatal monitoring, increased births attended by a skilled provider, greater health facility deliveries, mixed results on fertility, and decreased LBW risk [28].

While the evidence on dose-response impacts of CTs on health outcomes is limited, there are studies that support our approach and findings. In Brazil, a dose-response association was observed between the *Bolsa Familia Programme*, both in terms of cash amounts and program duration, and reduced maternal mortality, which was explained by prenatal care visits and case-fatality during delivery [29]. Relatedly, ANC was a posited mediator in our study given that LEAP 1000 was associated with an 11.4% point increase in ANC from a skilled provider during pregnancy [13]. Further, ANC is associated with improved birth outcomes and increased birthweight [30]. However, ANC was not shown to be a mediator in our study’s SEM analyses. These disparate findings may be explained by the differences in *Bolsa Familia* and LEAP 1000. *Bolsa Familia* is a conditional CT program that imposes ‘soft conditionalities’ on beneficiaries to attend prenatal care for continued payment whereas LEAP 1000 has no such conditions. Nonetheless, both studies demonstrate improvements in maternal health resulting from CTs.

We found no mediation by current NHIS enrollment or household food insecurity. Pregnant women enrolled in NHIS receive myriad services for free including maternity care [31]. These free healthcare services may then positively influence health-seeking behavior [32], which we found to be the case in the LEAP 1000 impact evaluation [13]. LEAP 1000 increased NHIS enrollment by 14.1% points and increased ANC utilization by 11.4% points overall [13, 14]. The null findings among our sample of infants may suggest the need to address additional supply- and demand-side barriers to NHIS annual renewal and health care utilization, such as the quality of health facilities in the area [33]. Furthermore, all pregnant women in Ghana were entitled to NHIS fee waivers, likely diluting the impacts of LEAP 1000 treatment on NHIS enrollment. Moreover, de Brauw and Peterman found robust impacts of El Salvador’s *Comunidades Solidarias Rurales* program on skilled attendance at birth and birth in health facilities, which they posit to be attributed to supply-side service improvements and enhancements in women’s agency [34], which inspired our assessment of women’s agency as a potential mediator but also points to care quality as a potentially important mediator that we did not explore in our study due to lack of data.

The absence of a mediating effect by household food insecurity can be explained, in part, by the intrahousehold, gendered dynamics that allocate household resources to men and boys as opposed to women and girls [35]. Moreover, household food security may not translate to or adequately capture individual nutrition and consumption behaviors. This may be a direct artefact of how household food insecurity was measured in this study – we use only two out of nine validated measures of food insecurity [36] to calculate our score as the nine items were assessed at endline only. Thus, our measure of household food insecurity may not adequately measure the true experiences of food insecurity in this sample.

The absence of a mediating effect by women’s agency conflicts with the literature. Women’s empowerment, which captures agency, is considered to be a salient factor in the improvement of maternal and child health outcomes [19, 21, 37] and has also been shown to be increased by CTs [17, 18]. Improvements in women’s empowerment translates to improved decision-making power which allows a woman to participate in decisions related to household resource and food allocation, healthcare seeking for themselves and their children, and demanding better quality of care from providers. We may not have been able to observe a mediating effect because of the way we defined women’s agency, which is notably difficult to define [38].

The LEAP 1000 impact evaluation offers a unique opportunity to conduct research on the dose-response relation between the number of months of program exposure before pregnancy and birthweight. LEAP 1000 is one of only a few CT programs in the African continent with primary objectives to reduce stunting in children under 5 years old that explicitly targets pregnant women, which is imperative to achieve program objectives. Evaluations of other programs that target pregnant women – Zambia’s Child Grant Program [39] and Mozambique’s Child Grant [40] – do not assess infant birthweight, which misses an important opportunity as birthweight is antecedent to stunting [41].

### Strengths

We used quasi-experimental, longitudinal data collected among pregnant and lactating women in high-poverty, rural Ghana to examine the dose-response associations between months of LEAP 1000 exposure and birthweight. This is the first study to examine duration of cash transfer exposure *in utero* and birthweight and also addresses the dearth of evidence of unconditional cash transfers and birthweight more broadly. Additionally, we use SEM, a statistical tool used to analyze complex relationships among variables [42], to explore mediators of these dose-response associations. We conduct sensitivity analyses to test our assumptions and appraise the validity of our main findings. The additional mediation analyses of this study provide useful information for program development and implementation as we highlight what is or isn’t working with program selection and operations that may be improved in future iterations.

### Limitations

This study has limitations that warrant discussion. In this sample of LEAP 1000 eligible women, only 60% reported delivering in health facilities and 50% of infants had a birthweight (either recorded on a health card or recalled by the mother). Our complete-case sample approach may result in biased findings owed to selection. The inclusion of infants born as part of multiple births and with weight recorded by maternal recall likely biased our results upward as multiple births generally have lower birthweights. Relatedly, we have a generally small sample that, though is well-powered to detect associations in linear regression, may be too small to reach statistical significance in the SEM models that use maximum likelihood estimation approaches. Further, the estimation of the effects of mediators on birthweight may still suffer from bias, as these are simultaneously assessed with effects of treatment on mediators in SEM. Also, issues of residual and uncontrolled confounding are likely to bias our results as there are certain measures likely to confound the mediator-outcome association that we have not considered. Further, it is possible that the household- and maternal-level variables assessed as potential mediators do operate along the pathway between LEAP 1000 and birthweight, but we were unable to capture their effects due to how these variables were measured and defined.

In our study, only 10% of infants were exposed to any number of months of LEAP 1000 before delivery due to the time-consuming processes of registration (occurring in March 2015) and payment delivery (starting from September 2015). Also, pregnant and lactating women with an infant up to 12 months old were targeted, meaning women who were visibly pregnant (usually around 4 months) and those who have already given birth likely comprise most of our targeted sample, which explains the low exposure prevalence in this sample. There was also a 6-month delay between targeting and enrolment (March 2015) and the first cash transfer receipt (September 2015), therefore many women had already given birth before receiving cash. All these contributors to small sample size and limited exposure limit the power of our analyses. In epidemiology, birthweight is a hotly debated outcome that is often considered a nebulous outcome in the absence of gestational age [43], which was not collected in the impact evaluation. We also were unable, due to lack of data and/or lack of statistical power, to include other mediators worthy of examination such as maternal nutrition, energy expenditures, and WASH indicators. The null findings in the SEM for LBW indicate greater need for inputs that could present clinically meaningful improvements in maternal and infant health beyond what is already allocated by LEAP 1000.

## Conclusions

Our findings suggest that LEAP 1000 exposure before delivery can increase birthweight and lower the risk of LBW. An absence of mediation in our study may serve to inform future program development and data collection strategies to better measure potential mediators. However, the low percentage of births exposed to CTs suggests that increased efforts are needed to target women earlier in pregnancy (and roll out cash payments faster) or prior to conception for CT receipt. Given that the treatment households studied here have now been exposed to seven years of CTs, and more births will have occurred, additional follow-up surveys should be conducted to understand impacts on birthweight and mediators of impact among a larger sample than feasible in the current study.

## Electronic supplementary material

Below is the link to the electronic supplementary material.

**Additional file 1**: Supplementary Figure 1

**Additional file 2**: Supplementary Table 1

## Data Availability

The data used in this analysis are publicly available from the University of North Carolina Population Center (https://data.cpc.unc.edu/projects/13/ view# res_ 226).

## List of abbreviations

ANC: Antenatal care
CI: Confidence interval
CT: Cash transfer
CCT: Conditional cash transfer
ISSER: Institute for Social, Statistical, and Economic Research
IUGR: Intrauterine growth restriction
LEAP: Livelihood Empowerment Against Poverty
LBW: Low birthweight
MoGCSP: Ministry of Gender, Children, and Social Protection
NHIA: National Health Insurance Authority
NHIS: National Health Insurance Scheme
OR: Odds ratio
PMT: Proxy means test
PTB: Preterm birth
SE: Standard errors
SEM: Structural equation modeling
UCT: Unconditional cash transfer
UNICEF: United Nations Children’s Fund
