# Supplementary material for: The dose-response association between LEAP 1000 and birthweight – no clear mechanisms: a structural equation modeling approach
Source: BMC Pregnancy Childbirth. 2023 May 19;23:364. doi: 10.1186/s12884-023-05707-1 (PMC10197393; doi:10.1186/s12884-023-05707-1)
Supplement: Supplementary file 1 — Additional file 1: Supplementary Figure 1 [file 12884_2023_5707_MOESM1_ESM.docx]

|  |
| --- |
| 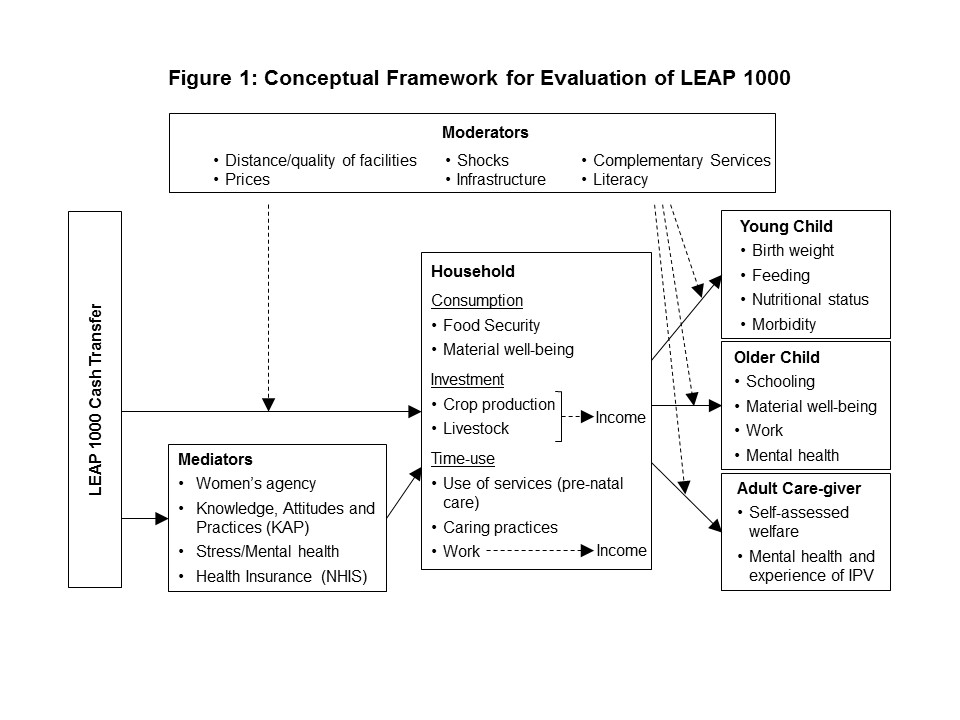 |
| **Supplementary Figure 1. Conceptual framework LEAP 1000 program evaluation; 2015-2017.**  **Source:** Ghana LEAP 1000 Evaluation Team. (2016). Ghana LEAP 1000 programme: baseline evaluation report. |
