# Supplementary material for: The dose-response association between LEAP 1000 and birthweight – no clear mechanisms: a structural equation modeling approach
Source: BMC Pregnancy Childbirth. 2023 May 19;23:364. doi: 10.1186/s12884-023-05707-1 (PMC10197393; doi:10.1186/s12884-023-05707-1)
Supplement: Supplementary file 2 — Additional file 2: Supplementary Table 1 [file 12884_2023_5707_MOESM2_ESM.docx]

| **Supplementary Table 1. Adjusted mediation effect of maternal and household-level characteristics on the association between months of LEAP 1000 treatment before delivery and LBW among the sample of 1,439 infants.** | | | | |
| --- | --- | --- | --- | --- |
| Variable  (1) | **Direct effect**  **(2)** | **Indirect effect**  **(3)** | **Percent due to mediation**  **(4)** |  |
| Months of LEAP 1000 | **-0.084 (-0.151, -0.018)**  **0.013** | 0.006 (-0.004, 0.016)  0.231 | 0.006/0.078 =7.7% |  |
| Women’s agency | -0.036 (-0.141, 0.068)  0.493 | -0.001 (-0.003, 0.002)  0.529 | 0.001/0.037 = 2.7% |  |
| Household food insecurity score (0-8) | -0.113 (-0.549, 0.023)  0.102 | 0.008 (-0.002, 0.017)  0.105 | 0.008/0.105 = 7.6% |  |
| Current NHIS enrollment | **-0.407 (-0.849, 0.036)**  **0.072** | -0.0001 (-0.003, 0.003)  0.961 | 0.0001/0.407 = <1% |  |
| ANC from a skilled provider | 0.406 (-0.474, 1.29)  0.366 | -0.001 (-0.002, 0.001)  0.605 | 0.001/0.405 = <1% |  |

ANC: Antenatal care; LBW: Low birthweight; LEAP 1000: Livelihood Empowerment Against Poverty 1000 program; NHIS: National Health Insurance Scheme; PMT: Proxy means test. Models adjusted for PMT score, parity, improved source of lighting in the household, number of children under 5 years old in the household, district of residence, year of birth, infant born in the rainy season, and month of birth. Standard errors clustered at household level. **Boldface results are those with a p-value less than 10%.**
